# *Lactobacillus reuteri* DSM 17938 Protects against Gastric Damage Induced by Ethanol Administration in Mice: Role of TRPV1/Substance P Axis

**DOI:** 10.3390/nu11010208

**Published:** 2019-01-21

**Authors:** Ana P. Oliveira, Luan K. M. Souza, Thiago S. L. Araújo, Simone de Araújo, Kerolayne M. Nogueira, Francisca Beatriz M. Sousa, Renan O. Silva, Dvison M. Pacífico, Conceição S. Martins, Gerly Anne de C. Brito, Marcellus H.L.P. Souza, Jand Venes R. Medeiros

**Affiliations:** 1 Laboratory of Pharmacology of Inflammation and Gastrointestinal Disorders (Lafidg), Federal University of Piauí, Av. São Sebastião, nº 2819, CEP 64202-02, Parnaíba, PI, Brazil; apatriciabiomed@gmail.com (A.P.O.); luankelves11@gmail.com (L.K.M.S.); thiago_parnaiba@hotmail.com (T.S.L.A.); simonearaujoufpi@gmail.com (S.d.A.); keerolayne@hotmail.com (K.M.N.); biatrix14@hotmail.com (F.B.M.S.); 2 Department of Physiology and Pharmacology, Federal University of Ceará, CEP 60430-270, Fortaleza, Ceará, Brazil; renan.oliveira25@yahoo.com.br (R.O.S.); souzamar@hotmail.com (M.H.L.P.S.); 3 Postgraduate Program in Morphofunctional Sciences, Department of Morphology, Faculty of Medicine, Federal University Ceará, CEP 60430-170, Fortaleza-CE, Brazil; dvisonpacifico@gmail.com (D.M.P.); concmartins@hotmail.com (C.S.M.); gerlyanneb@hotmail.com (G.A.d.C.B.)

**Keywords:** gastritis, alcohol, probiotic, TRPV, neurokinin

## Abstract

This study aimed to evaluate the effect of *Lactobacillus reuteri* DSM 17938 (DSM) on ethanol-induced gastric injury, and if its possible mechanism of action is related to inhibiting the transient receptor potential vanilloid type 1 (TRPV1). We evaluated the effect of supplementing 10^8^ CFU•g body wt^−1^•day^−1^ of DSM on ethanol-induced gastric injury. DSM significantly reduced the ulcer area (1.940 ± 1.121 mm^2^) with 3 days of pretreatment. The effects of DSM supplementation were reversed by Resiniferatoxin (RTX), TRPV1 agonist (3 nmol/kg p.o.). Substance P (SP) (1 μmol/L per 20 g) plus 50% ethanol resulted in hemorrhagic lesions, and DSM supplementation did not reverse the lesion area induced by administering SP. TRPV1 staining intensity was lower, SP, malondialdehyde (MDA) and nitrite levels were reduced, and restored normal levels of *antioxidant* parameters (glutathione and superoxide dismutase) in the gastric mucosa in mice treated with DSM. In conclusion, DSM exhibited gastroprotective activity through decreased expression of TRPV1 receptor and decreasing SP levels, with a consequent reduction of oxidative stress.

## 1. Introduction

Excessive alcohol consumption is an important public health problem, and globally, alcohol ingestion in 2016 was estimated to be 6.4 L of pure alcohol per person, which is related to risk of developing of several diseases, resulting in some 3 million deaths [1]. High ethanol concentrations present in alcoholic beverages have the capacity to directly injure the mucosa of the gastrointestinal tract, resulting in acute and chronic damage, such as erosive hemorrhagic gastritis and peptic ulcers [2,3,4]. Since ethanol is one of the most usually accepted and abused psychoactive drugs [1], the gastric injury generated by its abuse is a worrying consequence.

Acute ethanol administration disrupts the mucus–bicarbonate barrier, reduces antioxidant defenses, modulates the nitric oxide system, and reduces blood flow in the gastric mucosa [5,6,7,8]. An important mechanism by which ethanol mediates these harmful actions is through activating the transient receptor potential vanilloid 1 (TRPV1), which is present in the sensory nerves of the stomach [9]. Activating this receptor causes the release of Substance P (SP), which is capable of activating the neurokinin type 1 receptor (NK1) in gastric epithelial cells, and increases the generation of reactive oxygen species (ROS), the main cytotoxic agent of the gastric mucosa [9,10,11]. ROS cause peroxidation of lipid components in the cell membrane, and with increased amounts of these free radicals, together with antioxidant enzymes inhibition and glutathione depletion, ethanol causes cell damage as one of its main aggressive pathological mechanisms [12,13]. Thus, agents that inhibit the ethanol-mediated free radical generation pathway, such as TRPV1 and NK1 receptor antagonists, have a great potential to protect against ethanol-induced gastric mucosal lesions [9].

With regard to treating this type of injury, proton pump inhibitors are used, which may reduce the absorption of certain nutrients, in addition to other undesirable effects [14]. This motivates the search for optimized therapeutic agents for preventing and treating ethanol-induced injury, and in this context probiotics are potential candidates, because they present several beneficial activities in the gastrointestinal tract with few side effects [15]. In particular, lactic acid bacteria and their products are likely to contribute to the gastroprotective activity in ethanol-induced lesions, since they decrease levels of SP and lipid peroxidation [16,17,18,19]. However, there are still few studies on commercially available probiotic strains [20], such as *Lactobacillus reuteri* DSM 17938 (DSM) [21].

*L. reuteri* is a gram-positive, heterofermentative bacterium found in the gastrointestinal tract of humans and rodents [22]. This lactobacillus species has been used safely for many years, and many beneficial effects of the gastrointestinal tract have been attributed to DSM, such as reducing childhood colic, protecting against gastroenteritis, and ameliorating constipation and diarrhea [23,24,25,26]. The beneficial effects of this strain are mainly due to the production of antimicrobial compounds and stimulation of the immune system [27]. In addition, a recent study demonstrated that the DSM strain exhibits antinociceptive activity via inhibiting TRPV1 receptor expression [28].

This study was designed to evaluate the effect of DSM on ethanol-induced gastric lesions, and to evaluate if its possible mechanism of action is associated to inhibiting TRPV1 and the consequent reduction of SP, thereby minimizing oxidative stress and gastric injury.

## 2. Materials and Methods

### 2.1. Materials

*L. reuteri* DSM 17938 (Biogaia AB, Stockholm, Sweden) was purchased from Aché Pharmaceutical Laboratories (São Paulo, Brazil). Ethanol, Substance P acetate salt hydrate, Resiniferatoxin (RTX), and non-peptide NK_1_ tachykinin receptor antagonist (WIN 62,577) were purchased from Sigma Aldrich (St. Louis, MO, USA). The other reagents were of analytical grade bought from standard commercial companies. One milligram of RTX was dissolved in 1 mL of 95% ethanol and stored at −20 °C. When needed, this solution was diluted in 0.9% saline to the necessary concentration. WIN 62,577 was dissolved in dimethyl sulfoxide (DMSO). The other compounds were dissolved in saline when needed.

### 2.2. Animals

Swiss mice (25–30 g) were maintained at temperature control of 22 ± 2 °C, under a 12-h light/12-h dark cycle, with free access to food and water. The procedures were executed as specified by the Guide for Care and Use of Laboratory Animals (National Institute of Health, Bethesda, MD, USA), and the Ethics Committee in Research of the Federal University of Piauí previously approved all experiments under protocol number 292/17.

### 2.3. Experimental Design on the Ethanol-Induced Gastric Mucosal Damage

The animals were randomized into groups (5–6 mice per group), with different treatments as follow: Control animals (healthy or injured) were pretreated with saline (0.9%) by gavage; the other 3 groups received 10^8^ CFU/g body wt/day of freeze-dried DSM by gavage for 3, 7, or 14 days, according to previous studies [19]. The mice were deprived of food for 20 h before the ethanol-induced gastric damage, but had free access to water. After pretreatment, the animals supplemented with DSM and the injured group received ethanol 50% (0.5 mL/25 g p.o.), and the healthy group was treated with saline. After 1 h, the animals were euthanized, their stomachs were removed and opened along the greater curvature, and the gastric damage was measured using ImageJ software (National Institutes of Health, Bethesda, MD, USA), according to Medeiros et al. [29]. Tissues of each stomach were immediately fixed in 10% formalin for subsequent histopathological analysis. Unfixed samples of gastric mucosa were stored at −80 °C for biochemical analysis.

### 2.4. Evaluation of the Role TRPV1 and SP in Mediating the Protective Effects of DSM on Ethanol-Induced Gastric Mucosal Damage

To evaluate the role of the inhibition of TRPV1 in the protective effects of DSM in gastric mucosa, the mice were supplemented with DSM for 3 days, and on the fourth day, the animals received an agonist of TRPV1, RTX (3 nmol/kg) orally (p.o.) alongside 50% ethanol treatment (0.5 mL/25 g), according to Tramontana et al. [30], to activate the receptor.

To evaluate the possible role of NK1 modulation in gastric protection, a lesion was induced with the intraperitoneal administration of 1 μmol/L per 20 g of SP acetate salt hydrate, an agonist of the NK1 receptor, immediately before ethanol exposure, according to Karmeli et al. [11]. This lesion was induced after the 3 days of DSM administration in mice, or 30 min after the intraperitoneal administration of the NK1 antagonist, WIN 62,577 (20 mg/kg i.p.). One hour later, gastric damage was evaluated as described previously.

### 2.5. Histopathological Analysis of Gastric Damage

Photomicrographs of gastric ulcer and scores analysis were done by viewing ulcers with the use of microscope 100× and 400× magnification. Gastric ulcer condition was evaluated by the scoring system according to Laine and Weinstein [31].

### 2.6. TRPV1 Immunohistochemistry in Gastric Tissue

The tissue was stained for antigen–antibody complexes using a peroxidase detection system (LSAB kit, DAKO). Sections were rinsed with tris-buffered saline (TBS) buffer and incubated with rabbit polyclonal TRPV1 antibodies diluted 1:1000 overnight at 4 °C. The tissue slides were washed, incubated with a biotinylated secondary antibody (1:400) in phosphate buffered saline/bovine serum albumin (PBS–BSA), washed again, and were incubated with an avidin–biotin–horseradish peroxidase conjugate (by Vectastain) and peroxidase substrate for analysis with the chromogen 3,3’-diaminobenzidine tetrahydrochloride (DAB), which provided the slides the typical brown color of the reaction. The slides were counterstained with Harris’s hematoxylin. Negative control sections were processed at the same time as described, but without the TRPV1 antibody [32].

### 2.7. Immunohistochemical Analysis

The images were acquired from a camera with an LAZ 3.5 acquisition system (LEICA DM1000, Wetzlar, Germany) coupled to a light microscope. The resulting microscopic images were subjected to background subtraction and color correction.

The staining intensity of TRPV1 was estimated with ImageJ v1.46 software, according to the qualitative and nonsubjective method described by Helps et al. [33]. Hematoxylin and DAB component vectors were separated in section images, with a final magnification of 100×, by the ImageJ Color deconvolution (H-DAB) plugin. This method has demonstrated a linear correlation between increasing antibody concentration and DAB weight (DABwt) [34]. The results are expressed as DABwt%.

### 2.8. Enzyme-Linked Immunosorbent Assay (ELISA) for SP

The SP content in gastric tissue was analyzed by an enzyme immunoassay using a commercially available enzyme immunoassay kit from Cayman Chemicals (Ann Arbor, MI, USA). Tissue preparation and analysis was performed as described by Ma et al. [35].

### 2.9. Malondialdehyde Levels

The concentration of malondialdehyde (MDA) in stomach sample homogenates was measured by the method described by Mihara and Uchiyama [36].

### 2.10. Nitrate/Nitrite Levels in Gastric Tissue

The level of nitric oxide (NO) was measured by quantifying the NO metabolites nitrate (NO_3_^−^) and nitrite (NO_2_^−^) in the gastric tissue, according to the method described by Green et al. [37].

### 2.11. Reduced Glutathione Assay

The concentration of reduced glutathione (GSH) in stomach tissues as nonprotein sulfhydryls was estimated using the technique described by Sedlak and Lindsay [38].

### 2.12. Superoxide Dismutase Assay

Superoxide dismutase (SOD) activity was measured using a modified spectrophotometric assay [39]. In this method, enzyme activity is calculated by the amount of SOD capable of inhibiting nitrite formation by 50%. For this, a glandular fragment from each stomach was homogenized in 1 mL/100 mg of tissue phosphate buffer (50 nM, pH 7.4). One hundred microliters of the homogenate were added to 1110 μL of phosphate buffer, 75 μL of l-methionine (20 mM), 40 μL of Triton X-100 (1% *v*/*v*), 75 μL of hydroxylamine chloride (10 mM), and 100 μL of ethylenediaminetetraacetic acid (EDTA) (50 μM). This solution was incubated in a 37 °C water bath for 5 min, then 80 μL of riboflavin solution (50 μM) was added and exposed to light for 10 min. From this solution, 100 μL of the sample was withdrawn, and another 100 μL of Griess reagent was added to wells, and after 10 min, the absorbance was read at 550 nm by spectrophotometry on an ELISA reader. In addition, the amount of total proteins was determined with a commercial Labtest kit. The results were expressed as uSOD/μg of protein.

### 2.13. Gastric Wall Mucus

The determination of mucus content was carried out as described by Corne et al. (1974) after ethanol-induced gastric ulcer [40].

### 2.14. Gastric Acid Secretion

The pylorus ligature method was used to evaluate gastric acid secretion [41]. Initially, mice were supplemented with DSM, saline, or 50% ethanol *p.o.* for 3 days. After 24 h of fasting, the mice were anesthetized intraperitoneally with a combination of xylazine hydrochloride (5 mg/kg) and ketamine (60 mg/kg). Saline was injected into the duodenal lumen in mice that received DSM, saline, or ethanol. In another group, omeprazole (5 mg/kg) was injected into the duodenal lumen as well. After 4 h, animals were euthanized, the stomachs were opened, and the gastric contents were collected. Total acidity of the gastric juice was evaluated with 0.01 N NaOH, using 2% phenolphthalein as an indicator, and the final volume and pH were directly measured on the mucosal side of the stomach.

### 2.15. Statistic Analysis

The results were expressed as mean ± standard error of mean (±S.E.M.). Statistical analysis was performed using GraphPad Prism statistical software, version 6.0 (GraphPad Software Inc., San Diego, CA, USA). Differences between groups were evaluated using Analysis of Variance (ANOVA) and the Student–Newman–Keuls post hoc test, when appropriate. Moreover, the Kruskal Wallis nonparametric test, followed by Dunn’s test, were used in histopathological analyses. Difference between groups were considered statistically significant when *p* < 0.05.

## 3. Results

### 3.1. Effect of DSM on Ethanol-Induced Gastric Damag

**Figure 1:**
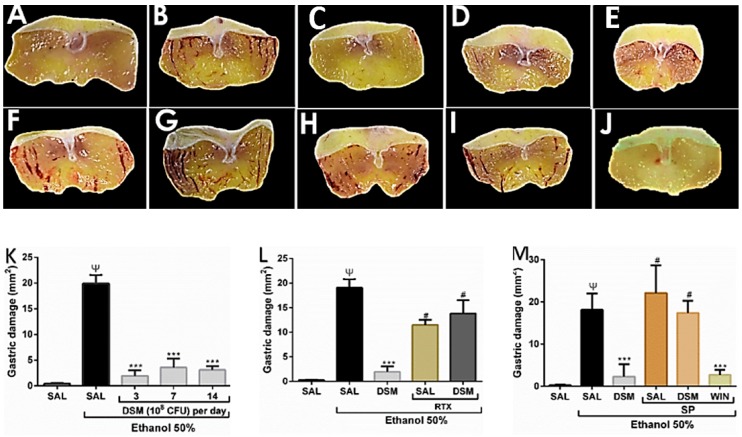
*Lactobacillus reuteri* DSM 17938 (DSM) supplementation on ethanol-induced gastric damage and the role of the TRPV1/substance P (SP) axis. (**A**): Healthy group (saline). (**B**): Injured group (50% ethanol). (**C**–**E**): Mice pretreated with 10^8^ CFU/g•bw/day DSM for 3 (**C**), 7 (**D**), and 14 days (**E**), followed by ethanol administration. (**F**): Mice treated with Resiniferatoxin (RTX, 3 nmol/kg p.o.) and 50% ethanol. (**G**): DSM Pretreatment (3 days) in combination with RTX and ethanol treatment. (**H**): Mice treated with 1 μmol/L per 20 g i.p. (SP) coadministered with 50% ethanol. (**I**): DSM supplementation in combination with SP and ethanol treatment. (**J**): Pretreatment with WIN-62577, an NK1 antagonist, in combination with SP and ethanol. (**K**): Macroscopic analysis of the gastric injuries upon treatment with ethanol alone and upon pretreatment with DSM. (**L**): Effects of treatment with RTX and ethanol (50%) in mice pretreated with DSM. (**M**): Effects of DSM supplementation on gastric damage induced by the combination of SP and ethanol treatment. The results are the mean ± S.E.M. of at least 5 mice per group. Results were analyzed using one-way analysis of variance (ANOVA) followed by a Newman–Keuls post hoc test. Ψ *p* < 0.0001 versus healthy group; *** *p* < 0.0001 versus injured group; # *p* < 0.0001 versus the DSM-supplemented group.

DSM supplementation significantly prevented ethanol-induced gastric injury. Figure 1 shows macroscopic photographs (B) and examinations (K) of mice treated with only 50% ethanol (0.5 mL/25 g *p.o.*), indicating significant gastric damage (19.920 ± 1.650 mm^2^), compared to healthy controls (A). DSM pretreatment for 3, 7, and 14 days protected the gastric mucosa from ethanol-induced injury (C, D, E, and K, respectively; *p* < 0.0001). Considering that 3 days of pretreatment with DSM significantly reduced the ulcer area (1.940 ± 1.121 mm^2^), all other experimental protocols to study the possible mechanisms of gastric protection were performed with 3 days of pretreatment.

### 3.2. Evaluation of the Possible role TRPV1 and NK1 Receptor in the Protective Effects of DSM on Ethanol-Induced Gastric Injury

To investigate the possible involvement of the TRPV1 receptor in DSM-mediated protection against ethanol-induced acute gastric lesions, the effect of treatment with the TRPV1 agonist, RTX (3 nmol/kg *p.o*.) with ethanol 50% in animals supplemented with DSM was evaluated. Macroscopic evaluation showed that animals treated with RTX alone and ethanol exhibited lesions of 11.480 ± 1.003 mm^2^ (Figure 1F,L), and the effects of DSM supplementation were reversed by RTX (13.800 ± 2.753 mm^2^) (Figure 1G,L).

To study the possible role of NK1 receptor inhibition in the gastroprotective effect of DSM, we assessed whether DSM was able to decrease the area of gastric lesions caused by the coadministration of ethanol and SP. SP (1 μmol/L per 20 g) plus 50% ethanol resulted in hemorrhagic lesions of 22.930 ± 2.930 mm^2^ (Figure 1H,M). DSM supplementation did not significantly reverse the lesion area induced by administering SP and ethanol (17.420 ± 1.280 mm^2^), as shown in Figure 1I,M. Treatment with the NK1 receptor antagonist, WIN-62577, did protect the gastric mucosa against the injury caused by coadministering SP and ethanol (Figure 1J,M; *p* < 0.0001).

### 3.3. Histopathological Parameters of DSM on Ethanol-Induced Gastric Damage

**Figure 2:**
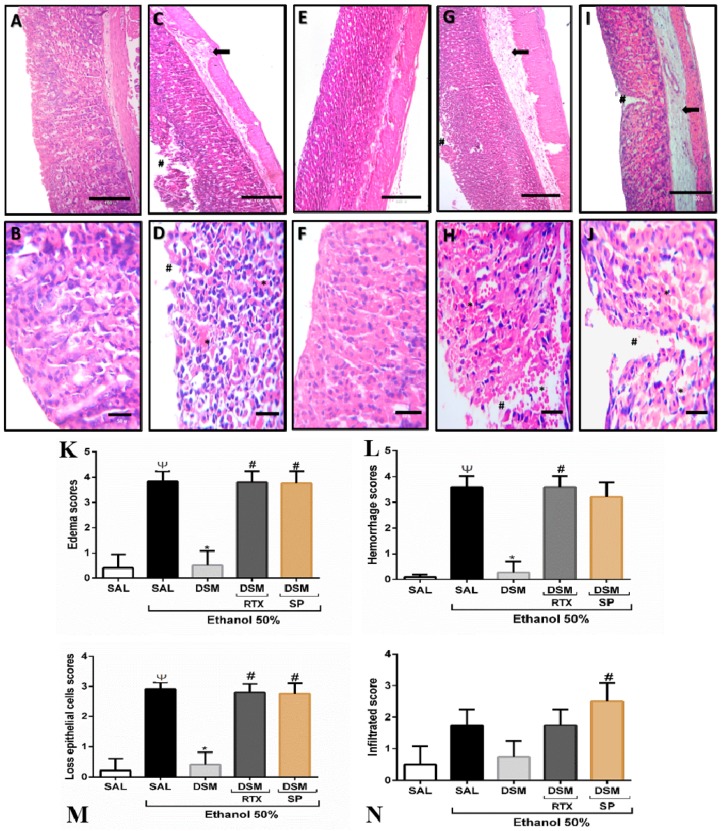
Histopathological analysis of gastric mucosa. (**A**–**J**) Photomicrographs: 100× magnification for the first line, and 400× for the second line. (**A**,**B**): Healthy group. (**C**,**D**): Injured group (50% ethanol). (**E**,**F**): DSM supplementation (10^8^ CFU/g•bw/day for 3 days) and ethanol administration. (**G**,**H**): Pretreatment with DSM (3 days) and treatment with RTX and 50% ethanol. (**I**,**J**): DSM supplementation and treatment with 1 μmol/L per 20 g i.p. of SP and 50% ethanol. Arrows: Edema; Tags: Epithelial cell loss; Asterisk: Hemorrhage. (**K**–**N**): Quantitative analysis of microscopic scores of the gastric mucosa upon ethanol-induced damage (hemorrhage, edema, epithelial cell loss, and infiltration). Results are expressed as mean ± S.E.M. (5–6 mice per group), and analyzed using Kruskal–Wallis nonparametric tests, followed by Dunn’s test. Ψ *p* < 0.05 versus healthy group; * *p* < 0.05 versus injured group; # *p* < 0.05 versus the DSM-supplemented group.

Histological evaluation confirmed that no damage was observed in the gastric mucosa of mice treated with saline alone (Figure 2A,B). Animals treated with 50% ethanol exhibited damaged gastric mucosa, loss of epithelial cells, edema, and enhanced hemorrhages (Figure 2C,D,K–N). DSM supplementation significantly inhibited these deleterious effects of ethanol in the gastric mucosa (*p* < 0.05, Figure 2E,F,K–N). Administering RTX with 50% ethanol in DSM-supplemented animals significantly reversed the protective effects of DSM in the gastric mucosa (Figure 2G,H,K–N). Pretreatment with DSM did not reduce the histopathological alterations resulting from the coadministration of ethanol and SP (Figure 2I,J,K–N). These microscopic results agreed with the macroscopic findings.

### 3.4. Immunohistochemistry Detection of the TRPV1 Receptor

**Figure 3:**
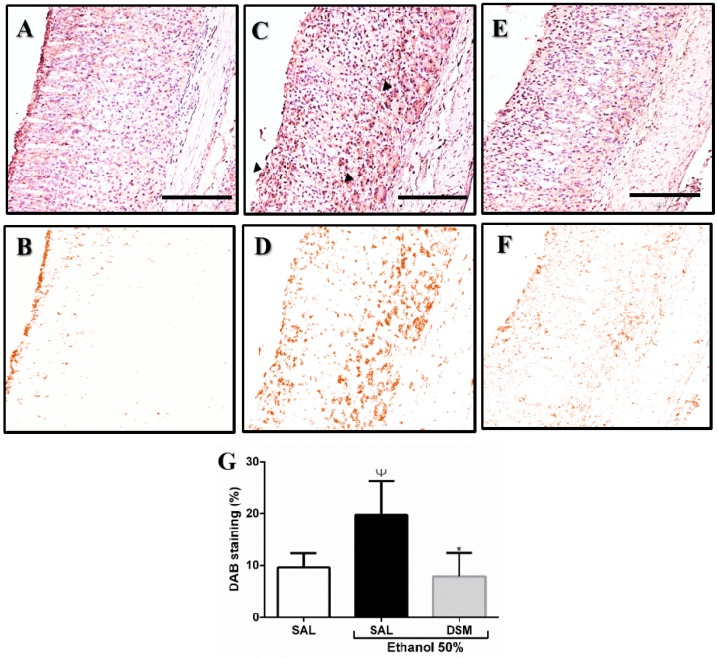
Immunohistochemical analysis of the TRPV1 receptor on the gastric mucosa (**A**,**C**,**E**). Diaminobenzidine (DAB) staining by color deconvolution (**B**,**D**,**F**). (**A**,**B**): TRPV1 immunoreactivity detected in normal gastric mucosa. (**C**,**D**): Increased immunoreactivity of TRPV1 in the ethanol-injured group. (**E**,**F**): Decreased immunoreactivity in gastric mucosa of animals pretreated with DSM for 3 days before ethanol-induced gastric damage. (**G**): Color deconvolution analysis of DAB staining for TRPV1 in gastric mucosa. One-way analysis of variance followed by a Newman–Keuls post hoc test. Ψ *p* < 0.05 versus healthy group; * *p* < 0.01 versus injured group. The arrows indicate the increase of marking intensity. Photomicrographs (100×, scale 100 µm).

The gastric tissues were examined immunohistochemically to determine the localization of the TRPV1 receptor (Figure 3). In the healthy control group (A and B), TRPV1 immunoreactivity was discreet, and the intensity of DAB staining was evident in the gastric mucosa upon ethanol treatment, with marked areas on the epithelium surface and submucosa (C and D). However, lower TRPV1 staining intensity was observed in mice treated with DSM (E and F). As shown in Figure 3G, the color deconvolution analysis of TRPV1 staining confirmed that ethanol treatment significantly increased DAB intensity (19.750 ± 2.903% DABwt), when compared to the saline and DSM groups (9.623 ± 1.404% DABwt; 7.922 ± 2.046% DABwt, respectively).

### 3.5. SP Levels

**Figure 4:**
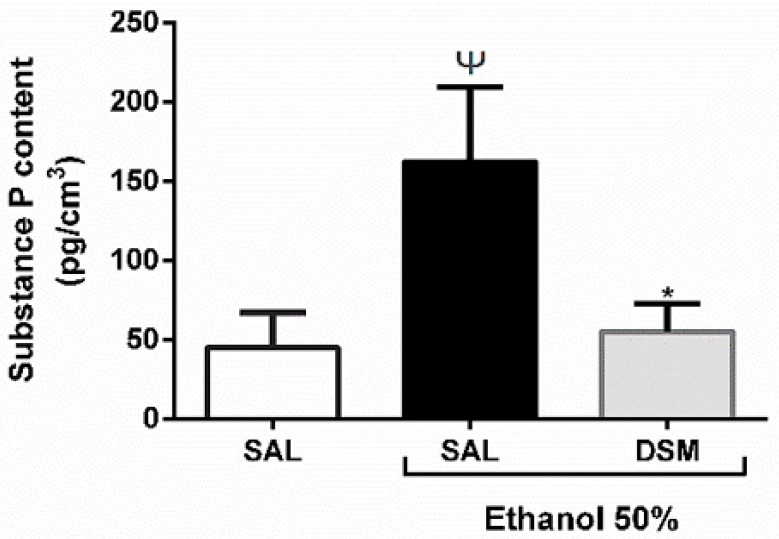
Effects of DSM supplementation on SP contents in gastric mucosa. The amount of SP in gastric mucosa was estimated by enzyme-linked immunosorbent assay (ELISA) and results were expressed as pg/cm^3^. Data shown are the mean ± SEM of at least 5 mice per group. The healthy group was treated with saline only. Ethanol administration significantly increased the gastric concentration of SP. This effect was reverted significantly when the animals were supplemented with DSM (10^8^ CFU/g•bw/day for 3 days). Results were analyzed using one-way analysis of variance (ANOVA) followed by a Newman–Keuls post hoc test. Ψ *p* < 0.05 versus healthy group; * *p* < 0.05 versus injured group.

SP levels were significantly reduced in mice pretreated with DSM compared to mice treated with ethanol alone (55.010 ± 7.827 pg/cm^3^ vs. 162.200 ± 21.090 pg/cm^3^ respectively; *p* < 0.05), as shown in Figure 4. These results suggested that DSM supplementation significantly decreased SP levels in gastric mucosa upon ethanol-induced gastric injury.

### 3.6. Effect of DSM on Biomarkers of Oxidative Stress and Antioxidant Parameters

**Figure 5:**
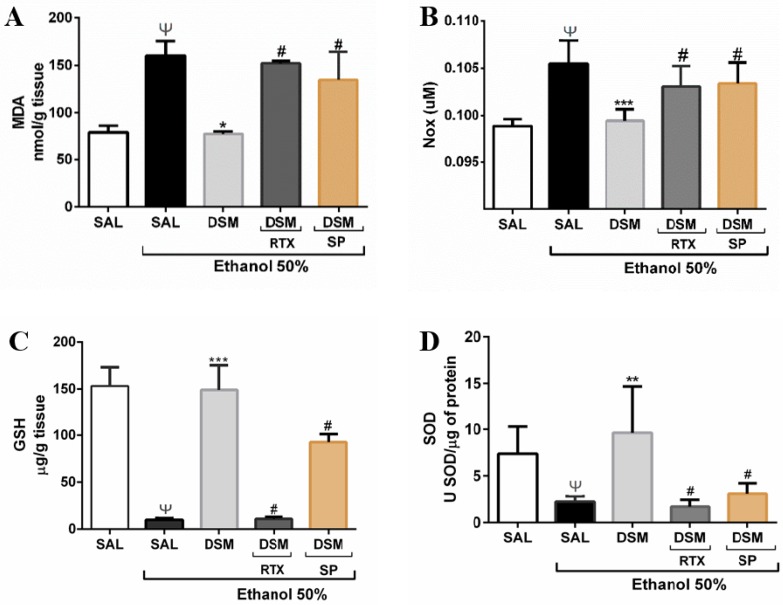
Effects of DSM supplementation on biomarkers of oxidative stress in mice with ethanol-induced gastric damage. Ethanol increased MDA (**A**) and NOx (**B**) levels in gastric tissue, but supplementation with DSM (10^8^ CFU/g•bw/day for 3 days) significantly prevented this effect. Ethanol administration decreased the gastric glutathione (GSH) concentration (**C**) and superoxide dismutase (SOD) activity (**D**). This effect was reverted significantly when the animals were supplemented with DSM. However, administration of RTX with ethanol reversed the protective effects of DSM for all parameters evaluated. Coadministering SP with ethanol overcame the protective effects of DSM. Data shown are the mean ± SEM of at least 5 mice per group; Results were analyzed using one-way analysis of variance followed by a Newman–Keuls post hoc test. (**A**): Ψ *p* < 0.05 versus healthy group; * *p* < 0.01 versus injured group; # *p* < 0.001 versus DSM group; (**B**): Ψ *p* < 0.0001 versus healthy group; *** *p* < 0.01 versus injured group; # *p* < 0.001 versus DSM group; (**C**): Ψ *p* < 0.0001 versus healthy group; *** *p* < 0.0001 versus injured group; # *p* < 0.0001 and # *p* < 0.05 versus DSM group; (**D**): Ψ *p* < 0.05 versus healthy group; ** *p* < 0.001 versus injured group; #*p* < 0.001 versus DSM group.

Malondialdehyde, a marker of oxidative stress and a product of lipid peroxidation, was measured to evaluate oxidative stress. Treatment with 50% ethanol increased the concentration of MDA in gastric mucosa, as shown in Figure 5A (160.400 ± 15.210 nmol/g). DSM supplementation significantly reduced the MDA levels in gastric damage induced by ethanol (74.940 ± 2.675 nmol/g). In addition, administering RTX with ethanol reversed the protective effect of DSM, and DSM did not decrease MDA levels upon coadministration of SP and ethanol (152.300 ± 2.425 nmol/g; 134.800 ± 29.710 nmol/g respectively; *p* < 0.001). Ethanol administration also increased NOx levels (Figure 5B), from levels observed in healthy animals and those pretreated with DSM (0.105 ± 0.001 µM; 0.097 ± 0.001 µM; 0.098 ± 0.001 µM, respectively). The treatment with RTX also reversed the protective effect of DSM on NOx levels, and DSM pretreatment did not reduce NOx upon SP treatment (0.103 ± 0.001 µM; 0.102 ± 0.001 µM; *p* < 0.001).

The GSH concentrations in the gastric mucosa of the five groups are shown in Figure 5C. The normal, baseline level of GSH in the gastric mucosa of animals was 153.300 ± 20.060 μg GSH/g tissue. The GSH concentration was significantly decreased (9.923 ± 1.931 μg GSH/g) upon administering 50% ethanol. DSM supplementation maintained the GSH concentration at basal levels (149.100 ± 26.330 μg GSH/g). RTX administration with ethanol significantly reversed the protective effects of *L. reuteri*. By contrast, DSM did not protect the gastric mucosa upon SP treatment, with GSH reduction (*p* < 0.0001; *p* < 0.05 respectively). The SOD activity in healthy animals was 7.376 ± 1.300 uSOD/mg of protein, and ethanol treatment decreased the SOD concentration in gastric tissue (2.240 ± 0.247 μSOD/mg of protein). DSM increased SOD levels, and this effect was reversed upon RTX administration, and did not increase upon SP treatment (9.620 ± 2.918 µSOD/mg protein; 1.700 ± 0.331 SOD/mg of protein; 3.090 ± 0.556 μSOD/mg protein, respectively; *p* < 0.001).

### 3.7. Gastric Wall Mucus and Acid Secretion

As shown in Table 1, administering 50% ethanol decreased Alcian blue adhesion to the gastric wall mucus, when compared with the amount of dye adhesion in the saline control group (58.340 ± 8.941 μg/g tissue; 137.200 ± 16.580 μg/g tissue, respectively). On the other hand, DSM (10^8^ CFU/g•bw/day) inhibited this effect of ethanol and rescued gastric mucus adhesion (103.201 ± 6.110 μg/g tissue; *p* < 0.01).

Moreover, no significant changes were observed in gastric juice volume, pH, and total acidity, among mice supplemented with DSM, omeprazole, or saline (Table 1). However, ethanol treatment increased these parameters, when compared to levels observed upon saline treatment alone (*p* < 0.01).

## 4. Discussion

The beneficial effects of probiotics in the GI tract have been demonstrated for various disorders [19,27,42,43]. In this study, we investigated the effects of *Lactobacillus reuteri* DSM 17938 supplementation on the protection of gastric damage induced by 50% ethanol in mice. Furthermore, we explored the role of the TRPV1/substance P axis in decreasing biomarkers of oxidative stress in gastric mucosa.

DSM, a widely recognized clinical and commercially available Lactobacillus strain [21,26] derived from the *L. reuteri* strain ATCC 55730 exerts beneficial effects for various GI tract conditions, such as constipation, diarrheal disease, infantile colic, and gastroenteritis [23,24,25,26,44], and exhibits immunomodulatory activity [27]. Furthermore, the use of 10^8^ CFU of DSM for 8 weeks results in a high eradication rate of *Helicobacter pylori* in the gastric mucosa, the main risk factor for developing chronic gastritis [45]. DSM administered at 10^6^ CFU/g•bw/day modulates the host mucosal immune system and induces an anti-inflammatory effect in a mouse model of necrotizing enterocolitis [46]. The effects of DSM 17938 on the gastrointestinal tract, in addition to the production of antimicrobial compounds such as reuterine, are mainly due to stimulation of the immune system through modulating dendritic cell responses [27]. In an experimental necrotizing enterocolitis model, this *L. reuteri* strain significantly reduces intestinal inflammation, inhibiting TLR4 and NF-KB receptor expression, in addition to modulating effector and regulatory T cells [46,47].

In our experiments, administering 10^8^ CFU/g•bw/day of freeze-dried DSM daily for 3, 7, or 14 days significantly prevented gastric damage induced by 50% ethanol treatment. These results are in accordance with recent data showing that lactic acid bacteria promote gastric protection against various harmful agents [17,18,19,48]. Rats pretreated with 2 × 10^9^ CFU/d of *Lactobacillus rhamnosus* GG for 3 consecutive days exhibited enhanced gastric mucosal integrity upon acute damage induced by ethanol [48]. Additionally, mice pretreated with 10^9^ CFU/kg•bw of *Lactobacillus fermentum* for 14 days also exhibited decreased HCl/ethanol-Induced gastric injury through its antioxidant effect [19].

In agreement with macroscopic findings, ethanol treatment directly injured the stomach mucosa, resulting in hemorrhagic lesions that caused edema, subepithelial hemorrhages, and cellular exfoliation with rupture of the mucosa [49]. In addition, treatment with *L. reuteri* significantly decreased these histopathological parameters, confirming its protective effect on the gastric mucosa. These results demonstrate an effect of DSM or its bacterial products on the gastric mucosa, which may have occurred because it presents acid and bile tolerance and mucus binding, facilitating its localization and protective action in the gastrointestinal tract [21].

Increased production of free radicals, such as reactive oxygen and nitrogen species, is related to the pathogenesis of ethanol-induced gastric mucosal damage, which damages important biomolecules, injures membranes, and causes cell death and epithelial loss [50]. These radicals cause peroxidation of the lipid components in the cell membrane, and the lipid peroxides are metabolized by β-oxidation to malondialdehyde, an important indicator of tissue damage by free radicals [13]. Moreover, increasing concentrations of these free radicals, along with inhibition of antioxidant enzymes and glutathione depletion, cause damage and cell death [51]. Gazzieri et al. [9] showed that activation of the TRPV1 receptor by ethanol in the nerve endings of the mucosa causes the release of SP and ROS generation, by a mechanism dependent on the TRPV1 receptor and the NK1 receptor, which mediates the effects of SP. Thus, substances that act as antagonists of these receptors have great potential for preventing and treating gastric lesions generated by alcohol.

Moreover, Perez-Burgos et al. [28] recently showed that DSM inhibits TRPV1 receptor expression, a mechanism involved in its antinociceptive activity in rodents treated with 1 × 10^9^ CFU for 9 days. Therefore, we evaluated the potential involvement of TRPV1 in the protective effect of DSM against ethanol-induced injury. For this, we activated the TRPV1 receptor with its specific agonist RTX in the gastric lesion in conjunction with ethanol treatment and found that RTX treatment blocked the gastroprotective activity of DSM. Histopathological findings corroborated these results. The immunohistochemistry results demonstrated decreased expression of this receptor in the gastric mucosa upon DSM supplementation, confirming our hypothesis that the presence of this bacterium may decrease the expression of TRPV1 activation by ethanol. In addition, an ELISA assay showed decreased substance P levels upon DSM supplementation. The literature also shows that other lactobacilli, such as *Lactobacillus casei* and *Lactobacillus fermentum*, inhibit the expression of TRPV1 and have intestinal therapeutic effect [52,53], and *L. fermentum* also decreases the levels of substance P to exert its gastroprotective effect [19].

The literature shows that the binding of SP to neurokinin type I receptor and its activation is necessary for the deleterious actions of this neurokinin, and that NK1 receptor antagonists have gastroprotective activity in the ethanol-induced injury [9,11]. We also assessed whether *L. reuteri* could modulate the SP receptor as a protection against ethanol action. Thus, as already described in the literature, administration of SP along with 50% ethanol increases the lesion caused by ethanol alone, and NK1 antagonists can effectively reverse the lesion generated [9]. However, in our study, pretreatment with DSM was not able to reverse the lesion caused by this agent, whereas the NK1 antagonist, WIN 62,577, significantly reduced the area of gastric lesion. Thus, DSM probably does not act by modulating this receptor in its gastroprotective effect. In this way, we suggest that the reduction of substance P levels in treated animals may be due to inhibition of TRPV1 receptor activation that this probiotic promoted in our study.

This study and previous studies showed that biomarkers of oxidative stress, such as levels of free radicals and MDA, are significantly increased by treatment with 50% ethanol [29,54]. DSM supplementation reduced levels of lipid peroxidation, causing a decrease in MDA concentration, and this protective effect was reversed upon treatment with a TRPV1 agonist. Pretreatment with DSM did not decrease MDA concentrations when SP was coadministered with ethanol to damage the gastric tissue, in agreement with previous results in this study. In our findings, ethanol increased the NOx levels, which are metabolites of nitric oxide (circulating nitrites and nitrates) in the gastric mucosa. DSM pretreatment maintained normal NOx levels, but this protective effect was reversed upon treatment with a TRPV1 agonist, and DSM could not reduce the NOx levels alongside SP administration. Previous studies show that acute ethanol administration increases neutrophil nitric oxide production through activating iNOS (inducible NO synthase), agreeing with our NOx results [55,56]. In addition, other studies with probiotics demonstrated decreased iNOS activation, and consequently, nitrite levels, in the probiotics’ beneficial effects in different models [20,52,53].

An important defense mechanism that organisms possess for maintaining the gastric mucosa against free radicals is enzymatic and nonenzymatic antioxidant systems [57]. An imbalance between the protective and damaging factors in the gastric mucosa decreases the defense capacity of the stomach, leading to gastric injury [54]. Superoxide dismutase is a metalloenzyme that catalyzes the reaction that transforms superoxide anions into hydrogen peroxide, playing an important role in eliminating reactive oxygen species, and ethanol treatment decreases the activity of this enzyme [12,58]. In this study, ethanol administration decreased SOD levels, and the pretreatment with DSM increased SOD activity despite ethanol-induced gastric damage. In addition, ethanol reduces the concentration of nonprotein sulfhydryl groups by depleting glutathione, a protective factor of the gastric mucosa [59].

Another important antioxidant process occurs through glutathione peroxidase, which converts glutathione to oxidized glutathione, and this process reduces H_2_O_2_ to H_2_O and hydroperoxide lipids into stable alcohols, playing an important role in protecting cells against the effects of hydrogen peroxide. Glutathione reductase reduces oxidized glutathione to GSH [58,60]. In agreement with results of previous studies, ethanol decreased GSH concentration in the gastric mucosa, and supplementation with DSM maintained normal levels of this protective mucosal factor of the mucosa upon gastric injury induced by ethanol.

The activation of the TRPV1 receptor with its ultrapotent agonist, RTX, overcame the protective effect of DSM pretreatment on levels of SOD and GSH in gastric tissue, showing that these antioxidant factors are also related to the action of DSM via TRPV1. However, DSM did not inhibit the depletion of antioxidant factors induced by coadministering SP and ethanol. These results reinforce our hypothesis that decreased oxidative stress and increased gastric protection by DSM in ethanol-induced gastric injury occurred through the TRPV1 receptor, and not through the receptor of SP.

Mucus secretion and gastric acid production are important homeostatic factors that maintain mucosal integrity, and an imbalance in this homeostasis can lead to ulceration [61,62,63]. The literature shows that excess alcohol can significantly reduce the layer of protective mucus, reducing its ability to defend against aggressive agents [64]. In this study, ethanol was reduced the mucus layer of the gastric wall, and pretreatment with DSM prevented this mucus reduction in ethanol-injured animals.

Studies show that levels of SP are elevated in gastric lesions and that tachykinin may increase gastric acid secretion, which can aggravate the lesions, since it is an excitatory gastrointestinal hormone [19,65]. In our study, as already discussed, ethanol increased levels of SP, and also increased acid secretion. Furthermore, supplementation with DSM did not appear to modify the normal volume of gastric secretion, pH, or total acidity in gastric mucosa of mice.

## 5. Conclusions

In summary, our findings showed that *L. reuteri* DSM 17938 supplementation protected the gastric mucosa from ethanol-induced gastric injury in mice. This activity was mediated by reducing oxidative stress and maintaining antioxidant activity, despite decreased SP levels. Our results supported the hypothesis that DSM may act by decreasing TRPV1 receptor expression, inhibiting the effects of ethanol-mediated TRPV1, thereby decreasing reactive oxygen species levels in the gastric mucosa. Future studies are required to confirm the model generated in this work.

## Figures and Tables

**Table 1:** DSM supplementation (10^8^ CFU/g.bw/day for 3 days) on mucus levels and gastric acid secretion in mice.

Treatment	Volume (mL)	pH (Units)	[H+] (mEq/mL/4 h)	Mucus Levels (ug/g Tissue)
**Saline**	0.028 ± 0.02	5.6 ± 0.2	0.14 ± 0.02	137.20 ± 16.58
**Ethanol**	0.350 ± 0.08 ^Ψ^	3.5 ± 0.2 ^Ψ^	3.70 ± 0.83 ^Ψ^	58.34 ± 8.95 ^Ψ^
**Omeprazole**	0.046 ± 0.02 ***	5.2 ± 0.5 *	0.20 ± 0.19 *	-
**DSM**	0.055 ± 0.02 **	4.6 ± 0.5 *	0.12 ± 0.05 *	103.20 ± 6.11 **

^a^ Data shown are the mean ± SEM of at least 5 mice per group. ^Ψ^ *p* < 0.01 versus saline group; * *p* < 0.05, ** *p* < 0.01, *** *p* < 0.001 versus ethanol group. One-way analysis of variance followed by a Newman–Keuls post hoc test. *Lactobacillus reuteri* DSM 17938 (DSM).
